# An air door opening and closing time identification and stage division method based on the wind speed data of a single sensor

**DOI:** 10.1038/s41598-024-59334-7

**Published:** 2024-04-14

**Authors:** Ying Song, Shan Li, Wentian Shang

**Affiliations:** 1 School of Management Science and Engineering, Shandong Technology and Business University, Yantai, 264005 China; 2 College of Safety Science and Engineering, Liaoning Technical University, Huludao, 125105 China

**Keywords:** Applied mathematics, Energy infrastructure

## Abstract

In mines, tunnel ventilation is monitored using wind speed sensors to measure the stability of the mine ventilation system. However, opening and closing the air door will cause violent fluctuations in the monitoring data of the wind speed sensors. When false alarms are triggered, the staff can diagnose only the mine ventilation system based on their experience. A numerical simulation method is adopted to explore the changes in the flow field during the opening and closing of the air door to address this issue. In addition, a method that is based on the wind speed data of a single sensor is proposed to identify the time and divide the stages of air door opening and closing. The experimental results showed that the proposed method can successfully identify the air door opening and closing time and apply stage division when needed.

## Introduction

In mine ventilation systems, air doors are commonly built in tunnels to control the airflow direction and speed to maintain the airflow distribution in conformance with the designed route^1,2^. However, during the process of opening and closing the air door in the tunnel, the flow field at the air door’s location and the associated tunnel will change drastically, resulting in large fluctuations in the wind speed sensor monitoring data for the tunnel^3^. The fluctuations in the wind speed during this process are greater than those during other activities, such as car operation^4^. Large data fluctuations can cause wind speed sensor alarms, which interfere with normal mine production activities, making the application of mine ventilation management systems extremely difficult^5–8^. Therefore, it is crucial to identify the time duration and classify the air door opening and closing stages by monitoring the wind speed sensor data.

The essence of wind speed monitoring data is time series data. Therefore, fluctuations in the wind speed sensor monitoring data, which are caused by opening and closing the dampers, can be considered time series anomalies. In recent years, time series anomaly identification methods have been applied in many fields^9,10^. In blast furnace ironmaking, Zhou et al.^11^ proposed a method that integrates principal component analysis (PCA) and independent component analysis (ICA) to monitor and diagnose furnace anomalies during blast furnace ironmaking by using operational data from the BF ironmaking process. In semiconductor fabrication, Hsu et al.^12^ developed a multiple time series convolutional neural network (MTS-CNN) model to distinguish between normal and abnormal wafers by collecting monitoring data from multiple sensors. In oil and gas extraction, Soriano-Vargas et al.^13^ studied a visualization and analysis method that is based on an interactive visualization of the time series data for anomaly detection through monitoring the data of oil and gas reservoirs. In aviation automation, He et al.^14^ used an anomaly detection and mitigation algorithm that is based on online subspace tracking to achieve high accuracy in anomaly detection and low error in data recovery through online unmanned aerial vehicle (UAV) flight data. In power quality signals, Rodriguez et al.^15^ adopted a recurrent long short-term memory (LSTM) recurrent neural network (RNN) for power quality disturbance classification through a deep convolutional autoencoder and stacking to identify and classify disturbances through power quality signals. In speech signal recognition, Cao et al.^16^ employed an urban noise identification method using convolutional neural networks (CNNs) to classify urban noise by utilizing the monitored acoustic signals within a city. In pipeline transportation, Zang et al.^17^ utilized a small leak detection method based on virtual sample generation (VSG) and unified feature extraction (UFE) techniques, which improved accuracy in detecting small leaks in pipelines through deep mining of basic information and machine learning model training.

In mine ventilation, several methods exist for the effective detection of fluctuations in wind speed sensor data. Huang et al.^18^ proposed a hybrid coded adaptive evolutionary strategy (ES) algorithm, which can identify the location of faults in a ventilation network and predict the range of changes in wind speed data using data from multiple wind speed sensors. Zhao et al.^19^ introduced a method for building a tunnel fault wind speed range library using a 0–1 sensitivity matrix and applied an improved support vector machine (SVM) method for fault diagnosis and localization. However, the above methods have three major limitations: (i) They cannot be used to obtain additional information about a fault after acquiring the reason or location of its occurrence. (ii) They are used only for permanent or prolonged failures such as tunnel collapse, broken air doors, or fan malfunction. Normal operation with minor variations in time, such as the normal opening and closing of dampers, cannot be recognized. (iii) Their performance is significantly influenced by sensor location.

The identification method for air door opening and closing proposed in this paper aims not only to determine the air door opening and closing state but also to explore and clarify the relationship between the air door opening and closing and the abnormal fluctuation data of the wind-velocity sensor data to conduct intelligent identification. To better identify fluctuations in wind speed data caused by the opening and closing of air doors, this paper uses image recognition to identify time series data, which is different from the above methods.

This article is organized into five sections, including the current section. In Section "Numerical simulation", a numerical simulation is used to explore the air door opening and closing process and identify the specific flow field changes in the associated tunnel after closing. In Section "The proposed time identification and stage division method", a method for identifying air door opening and closing times and stage division via data enhancement via multiscale sliding windows, the extraction of deep features from the data via DWT, the classification of the data samples via a classification model, and the correction of the identification results via a regression model are proposed. The advantages of the SVM and least absolute shrinkage and selection operator (LASSO) models are validated in this paper in terms of their accuracy in air door opening and closing time recognition and stage division compared to other classification and correction models through a significant number of experiments, as shown in Section "Experimental studies". Conclusions and future work are presented in the last section.

## Numerical simulation

In this section, several numerical simulations were conducted to explore the specific changes in the tunnel’s flow field during the opening and closing of the air door. The model contains four parts: (1) a brief description of the physical model is given in the first part, (2) the mesh generation method and the quality of the final generated mesh are discussed in the second part, (3) the selections of the solution model and the parameter settings for the numerical simulations are given in the third part, and (4) the numerical simulation results are presented, and the flow field laws are summarized in the fourth part.

### Physical model

**Figure 1:**
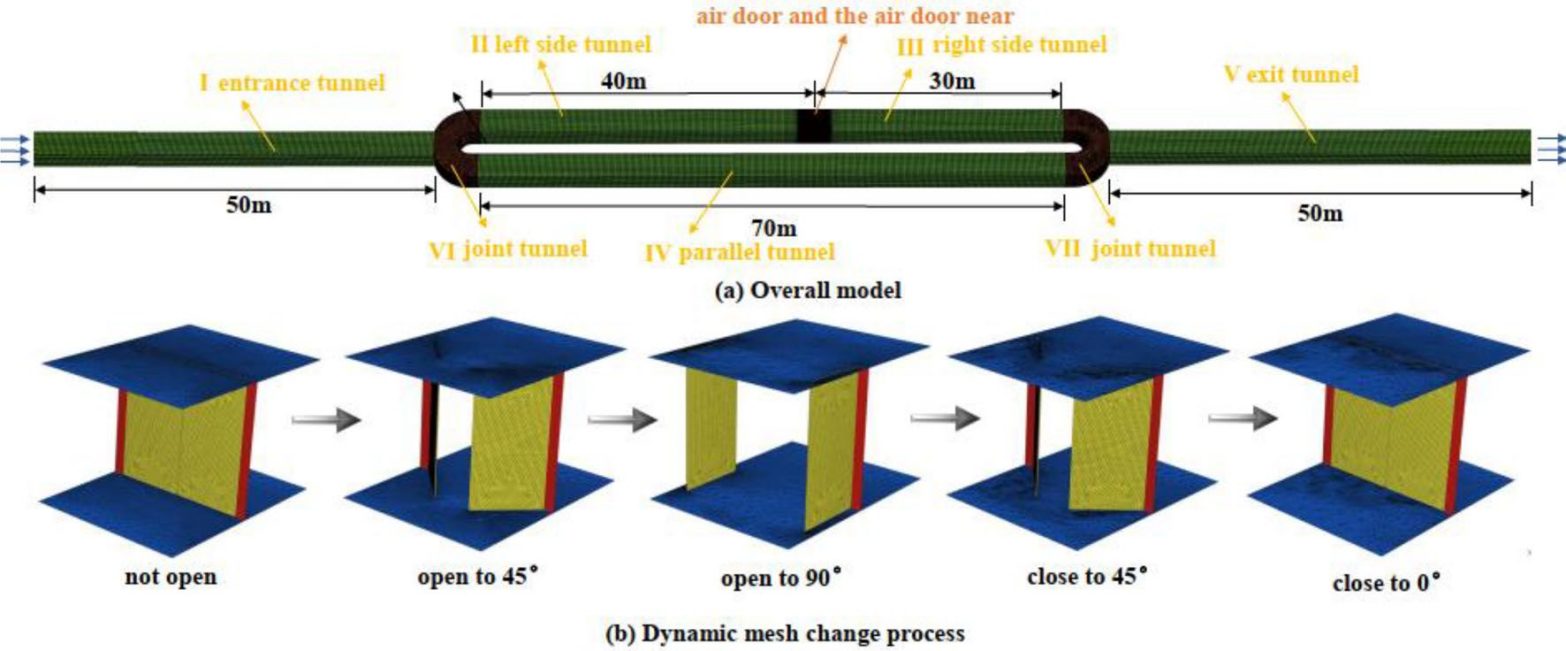
Physical model after meshing.

The numerical simulation model in this study refers to the experimental tunnel of the Laboratory of Mine Thermodynamic Disasters and Control of Ministry of Education. The tunnel section is rectangular, with a length of 2.5 m and a width of 3 m. The air door is located in the middle of the left and right sides of the tunnel. The detailed dimensions of the tunnel are shown in Fig. 1a.

### Mesh generation

Given the complex geometric structure of the physical model, partition and local encryption methods were used to divide the meshes. Tetrahedral meshes were used in areas VI and VII, with air doors within 5 m. Hexahedral meshes were used in areas I, II, III, IV, and V. Dense meshes were used within 5 m of the air door, and sparse meshes were used in the area.

After the grid dependency test, the final model in this study consisted of 434,700 meshes in the area within 5 m of the air door, 79,200 meshes in areas VI and VII, and 112,500 meshes in areas I, II, III, IV, and V, for a total of 626,400 meshes. Skewness is one of the quality inspection criteria of the mesh basic unit, where a value of 0 is the best quality and 1 is the worst quality. After the mesh division, the average skew of the entire model was approximately 0.283, and the maximum deflection of the mesh in the entrance area was 0.794. All of these values fall within the requirements of the mesh’s maximum deflection being less than 0.97 to ensure good mesh quality. The mesh generation results are shown in Fig. 1, where Fig. 1a shows the overall mesh of the model and Fig. 1b shows the mesh change process in the area within 5 m of the air door.

### Model selection and parameter setting

According to the actual mine ventilation system situation, the inlet and outlet boundaries are set as the velocity inlet and pressure outlet, respectively, and the physical quantities of the fluid within the physical model are set with reference to air. According to the literature^20–22^, the temperature is 20 °C, the humidity is 50%, the pressure is atmospheric pressure, the realizable k-ε turbulence model is selected as the solution model, and the coupled method with second-order upwind accuracy is selected for the solution. According to the air door motion law, the dynamic mesh is updated using the spring analogy model and the local redrawing model. After the time step sensitivity analysis, the selected time step is 0.0125 s.

### Numerical simulation results

**Figure 2:**
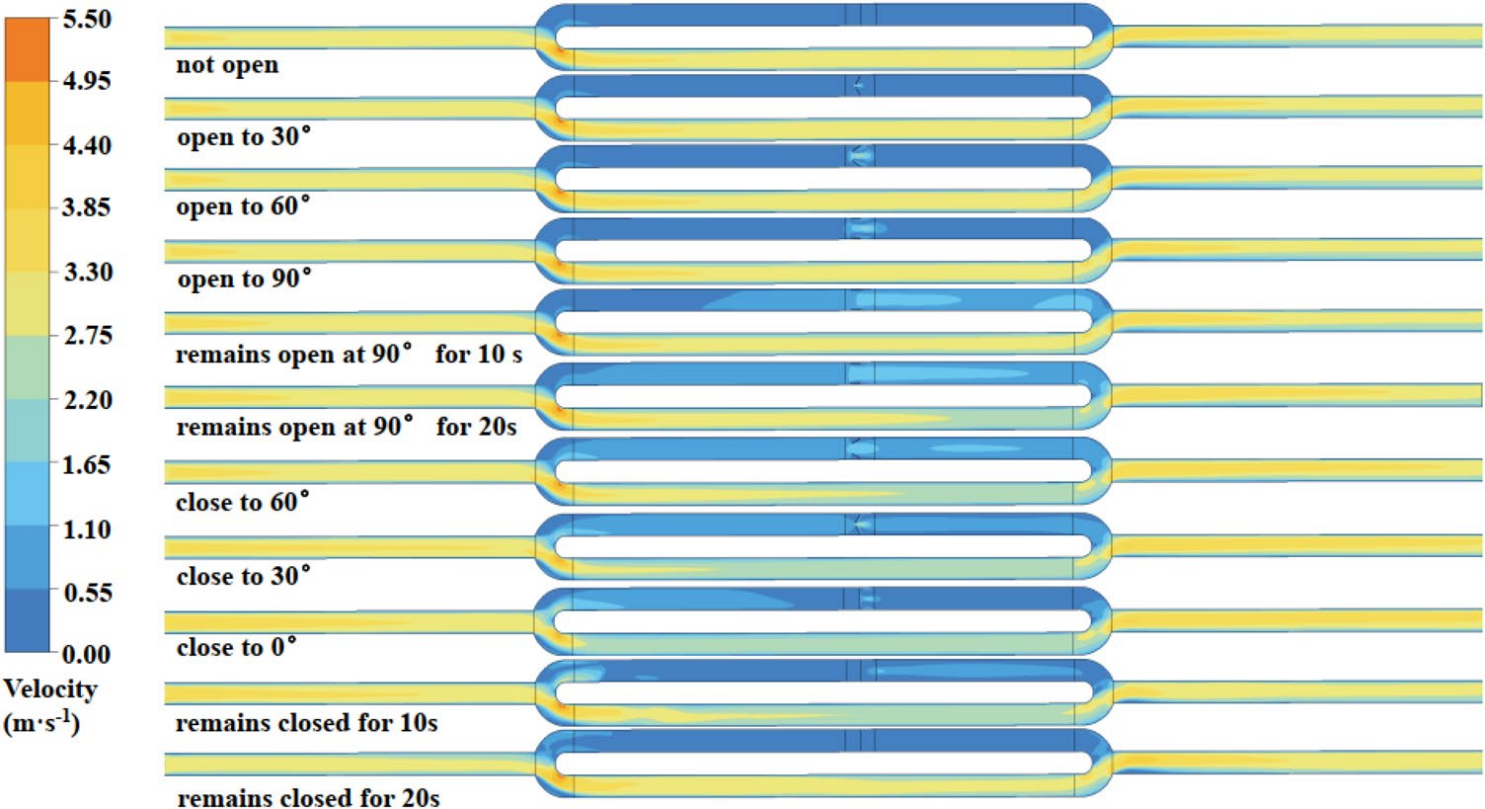
The flow field changes due to opening and closing the air door.

In this section, numerical simulations were conducted based on several condition parameters: (1) the inlet wind speed before opening the air door was 3 m/s; (2) the opening and closing angle was 90°; (3) the opening and closing speed was 15°/s; and (4) the time duration to open the air door to a fixed angle was 20 s. Figure 2 shows the numerical simulation results and the field velocity.

Figure 2 shows that a sudden increase in the wind speed develops during the opening and closing of the air door, where its influence is limited to the tunnel area where the air door is located. This is due to the narrower cross-section between the two doors compared to the tunnel section. Ultimately, the air door opens and closes the overall process, which dramatically changes the flow field. In the tunnel where the air door is located, the wind speed is significantly higher, and the flow field changes are more disturbing. In the tandem tunnel, the wind speed is slightly higher, and the flow field changes are more stable. In parallel tunnels, the wind speed is significantly lower, and the flow field changes are more stable. The flow field can still change even after the air door is close to 0°, after which it becomes stable.

## The proposed time identification and stage division method

This section describes the specific process of identifying the air door opening and closing time and the stage division method. It includes five parts. First, the overall architecture of the proposed time identification and stage division method is introduced. Second, the preprocessing step, including discrete normalization and multiscale sliding window discretization, is described in detail. Third, the classification steps, including feature vector extraction and the classification process, are described. Fourth, we introduce the merging and selection steps. Finally, the correction steps of the four regression models and their feature vectors are introduced.

### The architecture of the method

**Figure 3:**
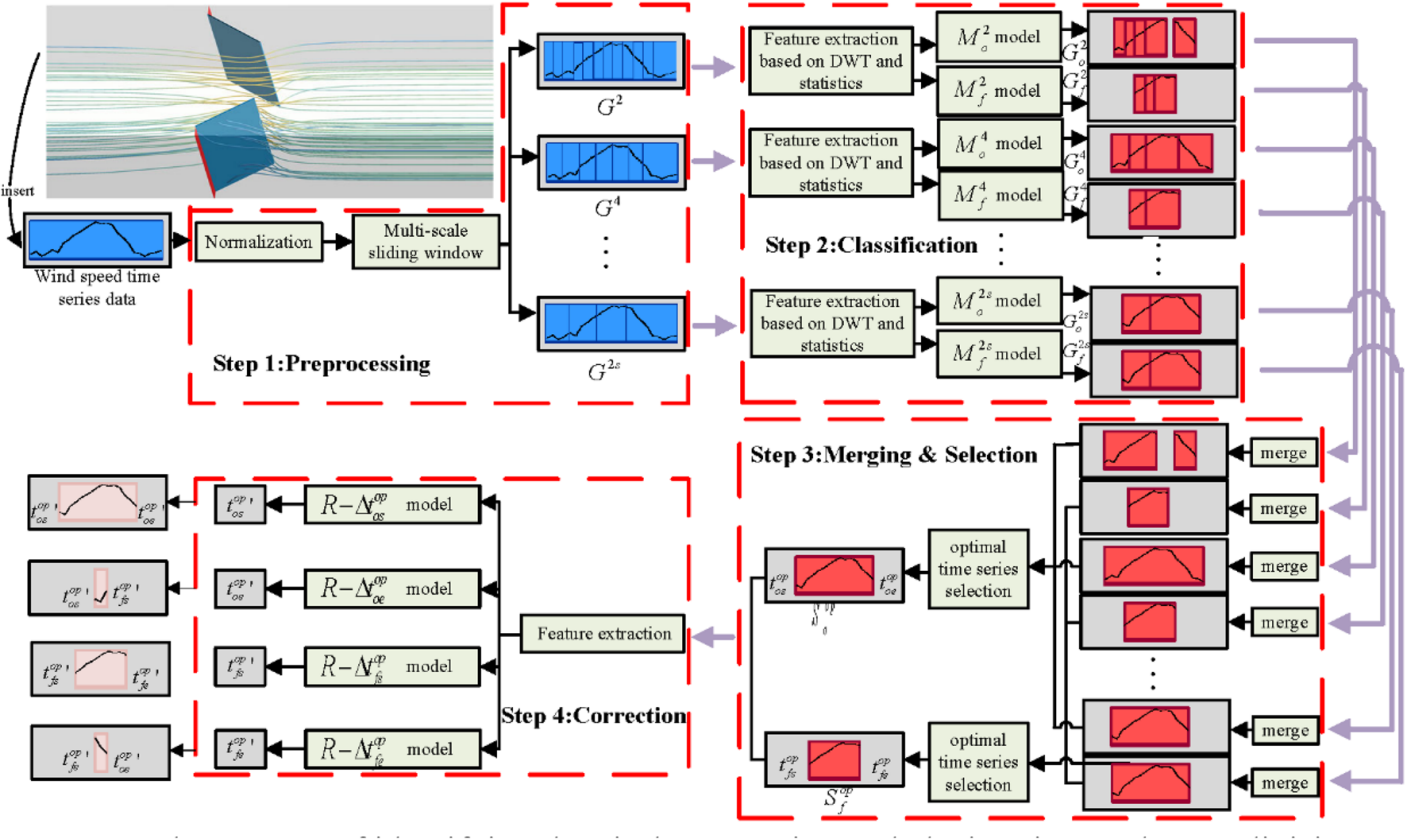
The process of identifying the air door opening and closing time and stage division.

Based on the research discussed in the previous two sections, the air door opening and closing time identification and stage division method is proposed based on the numerical simulation results. Figure 3 shows the architecture of the proposed method.

The proposed method includes four main steps, as follows:

Step 1: Preprocessing. The wind speed sensor data segment is processed into time series data with a varied range of [0, 1] using outlier normalization. Then, multiple original samples $\left\{ {G^{2} ,G^{4} , \ldots ,G^{2s} } \right\}$ composed of subtime series data are generated using the multiscale sliding window.

Step 2: Classification. The traditional statistics and discrete wavelet transform are used to extract the feature vectors from all the subtime series data of each original sample, and multimodels that are dedicated to this sample are used to complete the classification process. For example, in the original sample ($G^{k}$) classification process, one of the two classification models ($M_{o}^{k}$) is for classifying the air door opening and closing time, and the other ($M_{f}^{k}$) is for classifying the air door fixed angle opening time. In summary, two types of samples can be generated based on the original sample classification results. The first type is for the time identification samples $\left\{ {G_{o}^{2} ,G_{o}^{4} , \ldots ,G_{o}^{2s} } \right\}$ that are composed of different sets containing subtime series data that belong to the air door opening and closing category and its classification confidence. The second type is for stage division samples $\left\{ {G_{f}^{2} ,G_{f}^{4} , \ldots ,G_{f}^{2s} } \right\}$, which are composed of multiple sets containing subtime series data that belong to the air door’s fixed angle opening category and its classification confidence.

Step 3: Merging and Selection. All the subtime series data in each time identification sample or stage division sample are merged using the merge set approach. After the merging is completed for all the samples of both types, the optimal air door opening and closing time series data ($S_{o}^{op}$) and the optimal air door fixed angle opening time series data ($S_{f}^{op}$) are selected using the intersection over union (IoU) metric with classification confidence.

Step 4: Correction. Twelve basic features for the two optimal time series are extracted as the input features for using the four regression models $\left\{ {R - \Delta t_{os}^{op} ,R - \Delta t_{oe}^{op} ,R - \Delta t_{fs}^{op} ,R - \Delta t_{fe}^{op} } \right\}$. The two optimal time series starting and ending times $\left\{ {t_{os}^{op} ,t_{oe}^{op} ,t_{fs}^{op} ,t_{fe}^{op} } \right\}$ are corrected. Using the four correction times $\left\{ {t_{os}^{{op{\prime }}} ,t_{oe}^{{op{\prime }}} ,t_{fs}^{{op{\prime }}} ,t_{fe}^{{op{\prime }}} } \right\}$, the air door opening and closing times $\left[ {t_{os}^{{op{\prime }}} ,t_{oe}^{{op{\prime }}} } \right]$ can be identified and divided into the air door opening stage $\left[ {t_{os}^{{op{\prime }}} ,t_{fs}^{{op{\prime }}} } \right]$, air door fixed angle opening stage $\left[ {t_{fs}^{{op{\prime }}} ,t_{fe}^{{op{\prime }}} } \right]$, and air door closing stage $\left[ {t_{fe}^{{op{\prime }}} ,t_{oe}^{{op{\prime }}} } \right]$.

### Preprocessing

The wind speed sensor data are continuous time series data. Before processing the data using machine learning, data discretization and normalization are performed to obtain good classification performance^23–25^.

#### Dispersion standardization

Data normalization is an important preprocessing step that converts all the data of several ranges to fit in the range [0, 1], making different samples comparable^26^.

Normalization can be performed via various methods. In this study, deviation standardization is used to process the wind speed sensor data. The expression is shown in Eq. (1):

1 $$ x^{{\prime }} = \frac{{(x - x_{\min } )}}{{(x_{\max } - x_{\min } )}} $$

where $x^{{\prime }}$ represents the normalized data, $x$ represents the wind speed sensor data, $x_{\min }$ represents the minimum value of the wind speed sensor data, and $x_{\max }$ represents the maximum value of the wind speed sensor data.

The wind speed monitoring data $\left\{ {x_{1} ,x_{2} , \ldots ,x_{L} } \right\}$ change to $\left\{ {x_{1}^{{\prime }} ,x_{2}^{{\prime }} , \ldots ,x_{L}^{{\prime }} } \right\}$ after dispersion standardization is applied.

#### Multiscale sliding window discretization

**Figure 4:**
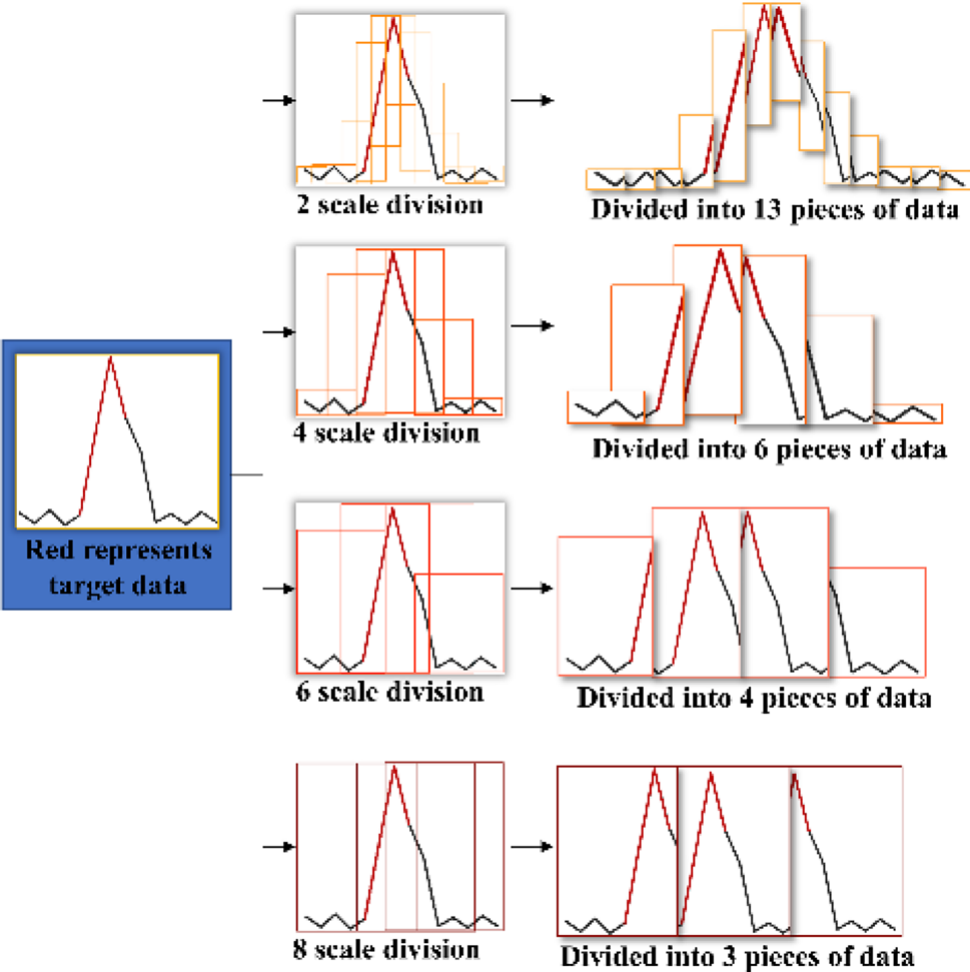
An example of continuous data discretized by a multiscale sliding window.

Discretization, which converts continuous data into discrete data with a finite number of intervals, is one of the most basic data partitioning techniques. Using discretized data allows for the construction of more efficient machine learning models^27–29^. Similar to most other time sequences, wind speed sensor data have multiscale properties. Figure 4 shows that the data at different scales can show different patterns. Therefore, the adopted discrete method in this study is the multiscale sliding window.

Based on the Coal Mine Safety Regulations in China and the data characteristics of the wind speed sensor data, the parameter constraints are obtained^4^. The parameters that are selected for the multiscale sliding window obey the following two constraints:

2 $$ P_{min} \le \{ W|w_{i} \} \le P_{max} $$

3 $$ \left\{ \begin{gathered} {\text{t}}_{i,k} = \frac{{w_{i} }}{2}\quad l_{i,k} \ge \frac{{w_{i} }}{2} \hfill \\ {\text{t}}_{i,k} = l_{i,k} \quad l_{i,k} < \frac{{w_{i} }}{2} \hfill \\ \end{gathered} \right. $$

Equation (2) is the constraint of the sliding window scale, where $W$ is the set of the sliding window scale, $w_{i}$ is the sliding window scale, and $P_{min}$ and $P_{max}$ represent the minimum and maximum values of a reasonable sliding window scale, respectively. When $P_{min}$ is greater than or equal to 2, $P_{max}$ is less than the quickest air door opening and closing time. Equation (3) is the constraint of the sliding distance, where ${\text{t}}_{i,k}$ is the sliding distance of the $w_{i}$ scale sliding window and $l_{{^{i,k} }}$ is the remaining length of the time series data after the $w_{i}$ scale sliding window has been slid $k$ times.

According to the two constraints of the multiscale sliding window, a segment of the normalized wind speed sensor data $\left\{ {x_{1}^{{\prime }} ,x_{2}^{{\prime }} , \ldots ,x_{L}^{{\prime }} } \right\}$ is discretized by a multiscale sliding window to generate multiple original samples $\left\{ {G^{2} ,G^{4} , \ldots ,G^{2s} } \right\}$. The expression of each sample is shown in Eq. (4):

4 $$ \left\{ {G^{k} |S^{k}_{i} } \right\},i = 1,2, \ldots ,n $$

where $G^{k}$ represents the sample discrete by the k-scale sliding window and $S^{k}_{i}$ represents the subtime series data within the *i*th k-scale sliding window. $S^{k}_{i} = \left\{ {x_{i \cdot k + 1}^{{\prime }} ,x_{i \cdot k + 2}^{{\prime }} , \ldots x_{i \cdot k + k}^{{\prime }} } \right\}$ when $i \ne n$; $S_{i}^{k} = \left\{ {x_{L - k}^{{\prime }} ,x_{L - 1}^{{\prime }} , \ldots ,x_{L}^{{\prime }} } \right\}$ when $i = n$.

### Classification

This section introduces two major points: the composition of the feature vectors extracted from each subtime series and the classification process for the specific flow.

#### Feature extraction method based on the DWT and statistics for the subtime series data

Statistical features can fully express the global information of subtime series data. Each subtime series statistical feature includes the mean value $\overline{{x^{{\prime }} }}$, the minimum value $x_{\min }^{{\prime }}$, the maximum value $x_{\max }^{{\prime }}$, and the standard deviation $\sigma$. The expressions of the features used in this study are listed in Table 1.

**Table 1:** Four statistical features.

Statistical features	Expression
Mean value	$\overline{x^{\prime}} = \frac{{\sum\nolimits_{i}^{k} {x^{\prime}_{i} } }}{k}$
Minimum value	$x^{\prime}_{min} = \min (S_{i}^{k} )$
Maximum value	$x^{\prime}_{max} = \max (S_{i}^{k} )$
Standard deviation	$\sigma = \frac{{\sum\nolimits_{i}^{k} {(x^{\prime}_{i} - \overline{x^{\prime}} )} }}{k}$

However, the limited statistical features cannot fully express the sample’s fluctuation information. A method for extracting fluctuation characteristics based on the DWT is proposed to mine the hidden information from subtime series data.

The DWT is a signal represented by a finite length or a fast-decaying oscillatory waveform that is scaled and panned to match the input data. It overcomes short-time Fourier transform (STFT) deficiencies with a good time–frequency local analysis capability and multiresolution analysis characteristics, which makes it widely used in signal and image recognition and detection. The process of a discrete wavelet transformation of the subtime series data can be expressed using Eq. (5):

5 $$ WT_{f} (q,w) = \int {_{R} } f(t) \cdot \overline{{\psi_{q,w} (t)}} \,dt $$

where $f(t)$ is subtime series data, $WT_{f} (q,w)$ is subtime series data resulting from discrete wavelet transform data, $t$ is the sequence of data, $q$ is a scale parameter, $w$ is the translation parameter along the time axis, and $\psi_{q,w} (t)$ is a wavelet base function. In this study, the db1 wavelet is used.

After processing by the DWT, the subtime series data are decomposed into several layers, each consisting of several high- and low-frequency coefficients. The fluctuation features are obtained by performing entropy sum calculations on the high- or low-frequency coefficients obtained from the multilayer decomposition. The entropy sum formula for any layer of the low- and high-frequency coefficients is calculated using Eq. (6):

6 $$ \left\{ \begin{gathered} e_{js} = \sum\limits_{i = 1}^{{n_{s} }} { - cs_{ji} \times \log_{2}^{{cs_{ji} }} } \hfill \\ e_{jd} = \sum\limits_{i = 1}^{{n_{d} }} { - cd_{ji} \times \log_{2}^{{cd_{ji} }} } \hfill \\ \end{gathered} \right. $$

where $e_{js}$ represents the entropy sum of the low-frequency coefficients in layer $j$, $e_{jd}$ represents the entropy sum of the high-frequency coefficients in layer $j$, $cs_{ji}$ represents the $i$ th low-frequency coefficient in layer $j$, $cd_{ji}$ represents the $i$th high-frequency coefficient in layer $j$, $n_{s}$ represents the total number of low-frequency coefficients in layer $j$, and $n_{d}$ represents the total number of high-frequency coefficients in layer $j$.

Since this article uses a db1 wavelet with a filter length of 2, the number of decomposition layers is calculated using Eq. (7):

7 $$ l = \left[ {\log_{2}^{k} } \right] $$

where $l$ represents the number of decomposable layers.

According to Eq. (6) and Eq. (7), the fluctuation features of each subtime series data are composed as shown in Eq. (8):

8 $$ \{ C_{f} |e_{is} ,e_{id} \} i = 1,2, \ldots ,l $$

where $C_{f}$ represents the set of fluctuation features, $e_{is}$ represents the entropy sum of the low-frequency coefficients in the *i*-layer, and $e_{id}$ represents the entropy sum of the high-frequency coefficients in the $i$-layer.

In summary, each subtime series feature vector consists of several statistical and fluctuating features. The subtime series data feature vector $C_{i}^{k}$ is shown in Eq. (9):

9 $$ C_{i}^{k} = \left( {\overline{{x^{{\prime }} }} ,x_{\min }^{{\prime }} ,x_{\max }^{{\prime }} ,\sigma^{2} ,e_{1s} ,e_{1d} , \ldots ,e_{Ls} ,e_{Ld} } \right)^{T} $$

To illustrate the feature vector clearly, Table 2 shows the composition of the feature vectors that are extracted by 2, 4, and 8 data lengths with the subtime series data covering the air door closing time and other times.

**Table 2:** Comparison of 2-, 4-, and 8-length target and non-target subtime series data features.

Data curve	Data length	Data time	$\overline{x}$	$x_{\min }$	$x_{\max }$	$\sigma^{2}$	$e_{1s}$	$e_{1d}$	$e_{2s}$	$e_{2d}$	$e_{3s}$	$e_{3d}$
	2	Other	0.500	0.442	0.558	0.082	0.048	0.500	–	–	–	–
	2	Air door closing	0.851	0.798	0.903	0.075	0.042	− 0.774	–	–	–	–
	4	Other	0.476	0.356	0.558	0.047	0.031	0.959	0.128	0.129	–	–
	4	Air door closing	0.834	0.798	0.903	0.050	0.050	− 1.332	0.011	− 4.110	–	–
	8	Other	0.404	0.308	0.558	0.101	0.041	1.919	0.136	0.650	0.191	− 0.501
	8	Air door closing	0.764	0.548	0.903	0.110	0.102	− 1.369	0.169	− 5.942	0.182	− 10.401

#### Classification process for the specific flow

Multiple models ($\left\{ {M_{o}^{2} ,M_{o}^{4} , \ldots ,M_{o}^{2s} } \right\}$ and $\left\{ {M_{f}^{2} ,M_{f}^{4} , \ldots ,M_{f}^{2s} } \right\}$) are used in the classification process. Their training and testing data can be found in Section "Experimental studies" of this article. Through the classification process, multiple time identification samples $\left\{ {G_{o}^{2} ,G_{o}^{4} , \ldots ,G_{o}^{2s} } \right\}$ and multiple stage division samples $\left\{ {G_{f}^{2} ,G_{f}^{4} , \ldots ,G_{f}^{2s} } \right\}$ can be obtained. This is an important prerequisite for air door opening and closing time identification and stage division. Algorithm 1 shows the procedure for the classification process.

**Algorithm 1:**
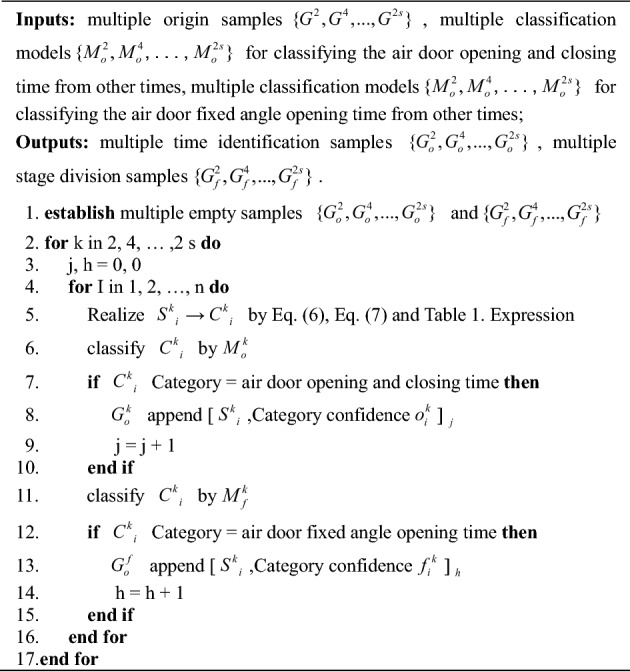
Classification process.

### Merging and selection

This section introduces two main processes. The first introduces the merging process of the subtime series. The second introduces the process of selecting the optimal time series based on the IoU and the confidence.

#### Subtime series merging

**Figure 5:**
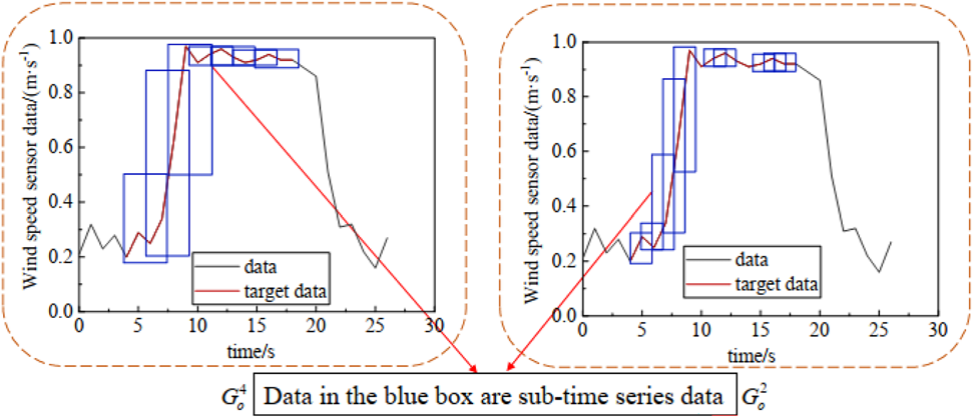
Example of different sample subtime series data before the merge.

Each time identification sample or stage division sample has many overlapping or nonoverlapping subtime series data, which are all part of the target time series that is selected by the classification model, as shown in Fig. 5. Therefore, we need to overlap the judgements and merge the two subtime series.

The IoU is used to determine whether two subtime series of data overlap. The IoU between two subtime series of data can be calculated using Eq. (10):

10 $$ IoU_{ab} = \frac{{S_{a}^{k} \cap S_{b}^{k} }}{{S_{a}^{k} \cup S_{b}^{k} }} $$

where $S_{a}^{k}$ and $S_{b}^{k}$ represent the two subtime series data.

When the IoU is greater than 0, the two time series data overlap. The union method is used to merge the two subtime series of data. The merging of the two overlapping subtime series data and the confidence calculation of the merged subtime series data are shown in Eq. (11):

11 $$ \left\{ \begin{gathered} S_{m}^{k} = S_{a}^{k} \cap S_{b}^{k} \hfill \\ c_{m}^{k} = \frac{{c_{a}^{k} + c_{b}^{k} }}{2} \hfill \\ \end{gathered} \right. $$

where $S_{m}^{k}$ represents the merged subtime series data, $c_{m}^{k}$ represents the confidence of the merged subtime series data, and $c_{a}^{k}$ and $c_{b}^{k}$ represent the confidence of $S_{a}^{k}$ and $S_{b}^{k}$, respectively.

Based on the overlapping judgement and merging of the two time series datasets, a merging method for each sample time series is proposed. Algorithm 2 shows the merging process of the multiple time identification samples $\left\{ {G_{o}^{2} ,G_{o}^{4} , \ldots ,G_{o}^{2s} } \right\}$ using this method. The merging process of the stage division samples $\left\{ {G_{f}^{2} ,G_{f}^{4} , \ldots ,G_{f}^{2s} } \right\}$ using this method is the same.

**Algorithm 2:**
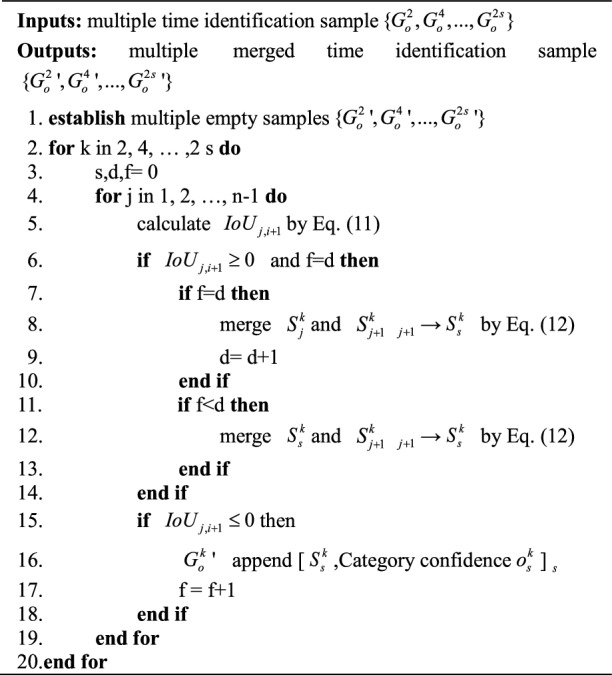
Time identification samples time series data merging process.

#### Optimal time series selection based on the IoU and the confidence

**Figure 6:**
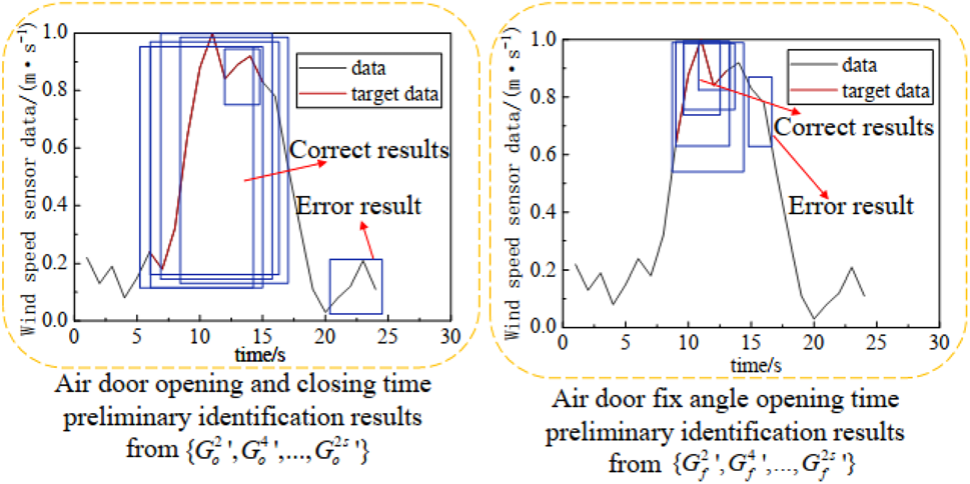
An example of the preliminary identification results.

For multiple merged identification samples $\left\{ {G_{o}^{{2{\prime }}} ,G_{o}^{{4{\prime }}} , \ldots ,G_{o}^{{2s{\prime }}} } \right\}$ or multiple merged stage division samples $\left\{ {G_{f}^{{2{\prime }}} ,G_{f}^{{4{\prime }}} , \ldots ,G_{f}^{{2s{\prime }}} } \right\}$, their subtime series data are considered preliminary identification results. These results include an error result and an accuracy gap between the correct results, as shown in Fig. 6. Therefore, an optimal time series selection method based on the IoU and the confidence interval is proposed.

In this method, the IoU threshold is used to eliminate incorrect results. Initially, an IoU threshold is set. If the average IoU value of a result is smaller than the threshold, the IoU value is eliminated. In this study, the IoU threshold is set to 0.1. After eliminating the error, the result with the highest confidence is selected as the optimal recognition result. The average IoU of each recognition result is calculated using Eq. (12):

12 $$ \overline{{IoU_{m}^{v} }} = \frac{{\sum\limits_{k = 1}^{{n_{k} }} {\left( {\sum\limits_{s = 1}^{{n_{s} }} {\frac{{S_{m}^{v} \cap S_{s}^{k} }}{{S_{m}^{v} \cup S_{{_{s} }}^{k} }}} } \right)} }}{{n_{s} \cdot n_{k} }} $$

where $\overline{{IoU_{m}^{v} }}$ represents the average IoU of the $S_{m}^{v}$ subtime series and the other subtime series data, $n_{s}$ represents the number of time series data in $G_{o}^{k}$ or $G_{f}^{k}$, and $n_{k}$ represents the number of time identification samples or stage division samples.

Using the optimal time series selection based on the IoU and the confidence method, the optimal air door opening and closing time series data ($S_{o}^{op}$) and its confidence ($c_{o}^{op}$) can be selected from the time identification samples $\left\{ {G_{o}^{{2{\prime }}} ,G_{o}^{{4{\prime }}} , \ldots ,G_{o}^{{2s{\prime }}} } \right\}$. The optimal air door fixed angle opening time series data ($S_{f}^{op}$) and its confidence ($c_{f}^{op}$) can be selected from the stage division samples $\left\{ {G_{f}^{{2{\prime }}} ,G_{f}^{{4{\prime }}} , \ldots ,G_{f}^{{2s{\prime }}} } \right\}$.

### Correction

Four regression models are established in this method. $R - \vartriangle {\text{t}}_{os}^{op}$ and $R - \vartriangle {\text{t}}_{oe}^{op}$ models with the start time correction $\vartriangle {\text{t}}_{os}^{op}$ and end time correction $\vartriangle {\text{t}}_{oe}^{op}$ of the optimal air door opening and closing time series data $S_{o}^{op}$. $R - \vartriangle {\text{t}}_{os}^{op}$ and $R - \vartriangle {\text{t}}_{oe}^{op}$ models with the start time correction $\vartriangle {\text{t}}_{fs}^{op}$ and end time correction $\vartriangle {\text{t}}_{fe}^{op}$ of the optimal air door opening and closing time series data $S_{f}^{op}$. In the training or testing of these regression models, the feature vector can be expressed using Eq. (13):

13 $$ C_{l} = \left( {t_{os}^{op} ,t_{oe}^{op} ,T_{o}^{op} ,\overline{x}_{o}^{op} ,\sigma_{o}^{op} ,c_{o}^{op} ,t_{fs}^{op} ,t_{fe}^{op} ,T_{f}^{op} ,\overline{x}_{f}^{op} ,\sigma_{f}^{op} ,c_{f}^{op} } \right)^{T} $$

where $C_{l}$ represents the feature vector, $T_{o}^{op}$ is the duration of the optimal air door opening and closing time series, $\overline{x}_{o}^{op}$ is the mean of the optimal air door opening and closing time series, $\sigma_{o}^{op}$ is the mean of the optimal air door opening and closing time series, $T_{f}^{op}$ is the start time of the optimal air door fix angle opening time series having a fixed angle, $\overline{x}_{f}^{op}$ is the mean of the optimal air door fix angle opening time series, and $\sigma_{f}^{op}$ is the mean of the optimal air door fix angle opening time series.

By applying four corrections for the time $\{ \Delta t_{os}^{op} ,\Delta t_{oe}^{op} ,\Delta t_{fs}^{op} ,\Delta t_{fe}^{op} \}$, the four times $\left\{ {t_{os}^{op} ,t_{oe}^{op} t_{fs}^{op} ,t_{fe}^{op} } \right\}$ are corrected as $\left\{ {t_{os}^{{op{\prime }}} ,t_{oe}^{{op{\prime }}} ,t_{fs}^{{op{\prime }}} ,t_{fe}^{{op{\prime }}} } \right\}$. The air door opening and closing time series data $S_{o}^{{op{\prime }}}$ can be located by $\left[ {t_{os}^{{op{\prime }}} ,t_{oe}^{{op{\prime }}} } \right]$. The air door opening stage $S_{oo}^{{op{\prime }}}$, air door fixed angle opening stage $S_{f}^{{op{\prime }}}$, and air door closing stage $S_{c}^{{op{\prime }}}$ can be characterized by $[t_{os}^{{op{\prime }}} ,t_{fs}^{{op{\prime }}} ]$, $[t_{fs}^{{op{\prime }}} ,t_{fe}^{{op{\prime }}} ]$, and $[t_{fe}^{{op{\prime }}} ,t_{oe}^{{op{\prime }}} ]$, respectively.

## Experimental studies

In this section, some experiments are conducted to verify the effectiveness of the proposed method. This section includes three parts: (1) the experimental system and data description, (2) the evaluation indices of the proposed method, and (3) experimental studies on the identification and division of air door opening and closing times.

### Experimental system and data description

This section is composed of two parts. The first part explains the principles of the experimental system design and the equipment configuration. The second part describes the experimental data.

#### Experimental system

The experimental model is designed according to the numerical simulation model and the flow similarity principle. To satisfy the geometric similarity between the experimental model and the original model, the overall similarity scale was taken as 1:16, and the rate of change in the length direction was taken as 2^30^.

**Figure 7:**
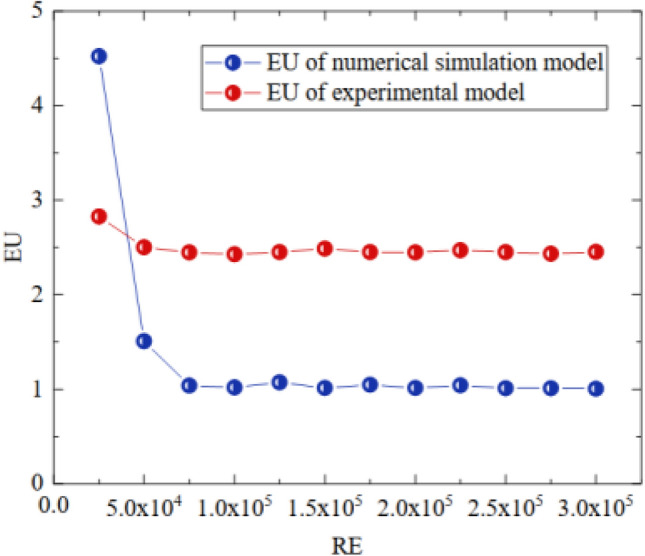
Curves of the variation in EU with respect to RE within the numerical simulation model and the experimental model.

According to the literature^30–32^, within two geometrically similar models, the flow field enters the second self-simulation zone when the Euler number (EU) is independent of the Reynolds number (RE), satisfying the flow similarity principle. We explored the similarity between the EU and RE by changing the wind speed to obtain the relationship between them in the experimental model and the numerical simulation model. Figure 7 shows the results of the EU with the RE within the experimental and numerical simulation models.

Figure 7 shows that the Euler numbers of both the experimental and numerical simulation models do not change when the Reynolds number is greater than 0.75 × 10^5^. Therefore, when the inlet wind speed is greater than 7.9 m/s in the numerical simulation model and greater than 0.49 m/s in the experimental model, the dynamics of the two flow fields can be considered similar.

**Figure 8:**
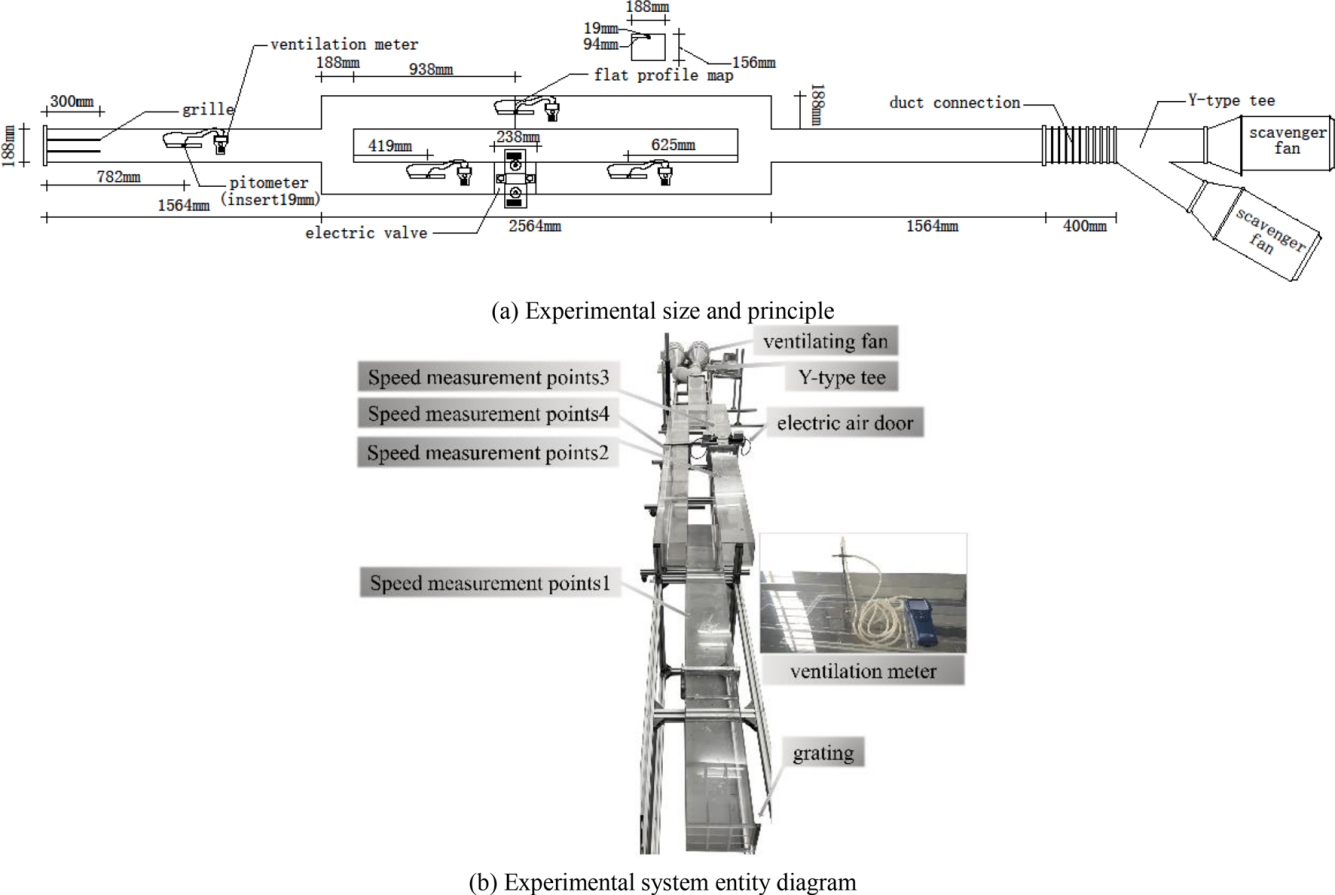
Experimental system.

According to the above, an experimental system with variable air door opening and closing parameters was designed, as shown in Fig. 8. Figure 8a shows the size and principles of the experimental system, whereas Fig. 8b is an entity diagram of the experimental system.

The experimental system included a parallel connection ventilation pipeline, an air supply system, a wind speed monitoring system, and an electric air door. The wind speed monitoring system was arranged according to the position regulations of the sensors in the mine. The parallel connection ventilation pipeline was made using a 4.8 mm thick acrylic board. The air supply system consisted of two ventilation fans connected in parallel, with a maximum ventilation capacity and rotating speed of 2206 m^3^/h and 2350 r/min, respectively, for every fan. The wind speed monitoring system is composed of four ventilation meter (model TSI-9565). The electric air door’s maximum opening angle was 90°, and the fastest opening and closing speed was 15°/s.

#### Experimental data

There were four variable parameters for the experimental system: inlet wind speed, air door opening and closing speed, air door opening and closing angle, and air door fixed angle opening time. Under the premise that the air door starts opening at 10 s, experiments were carried out for 240 working conditions by arranging and combining the different values of the four parameters, as shown in Table 3.

**Table 3:** Specific parameters of each component of the air door opening and closing factors.

Parameter	Values
1	3°/s, 5°/s, 10°/s, 15°/s
2	5 s, 10 s, 20 s, 30 s, 40 s
3	45°, 60°, 75°, 90°
4	8.5 m/s, 9.5 m/s, 10.5 m/s

**Figure 9:**
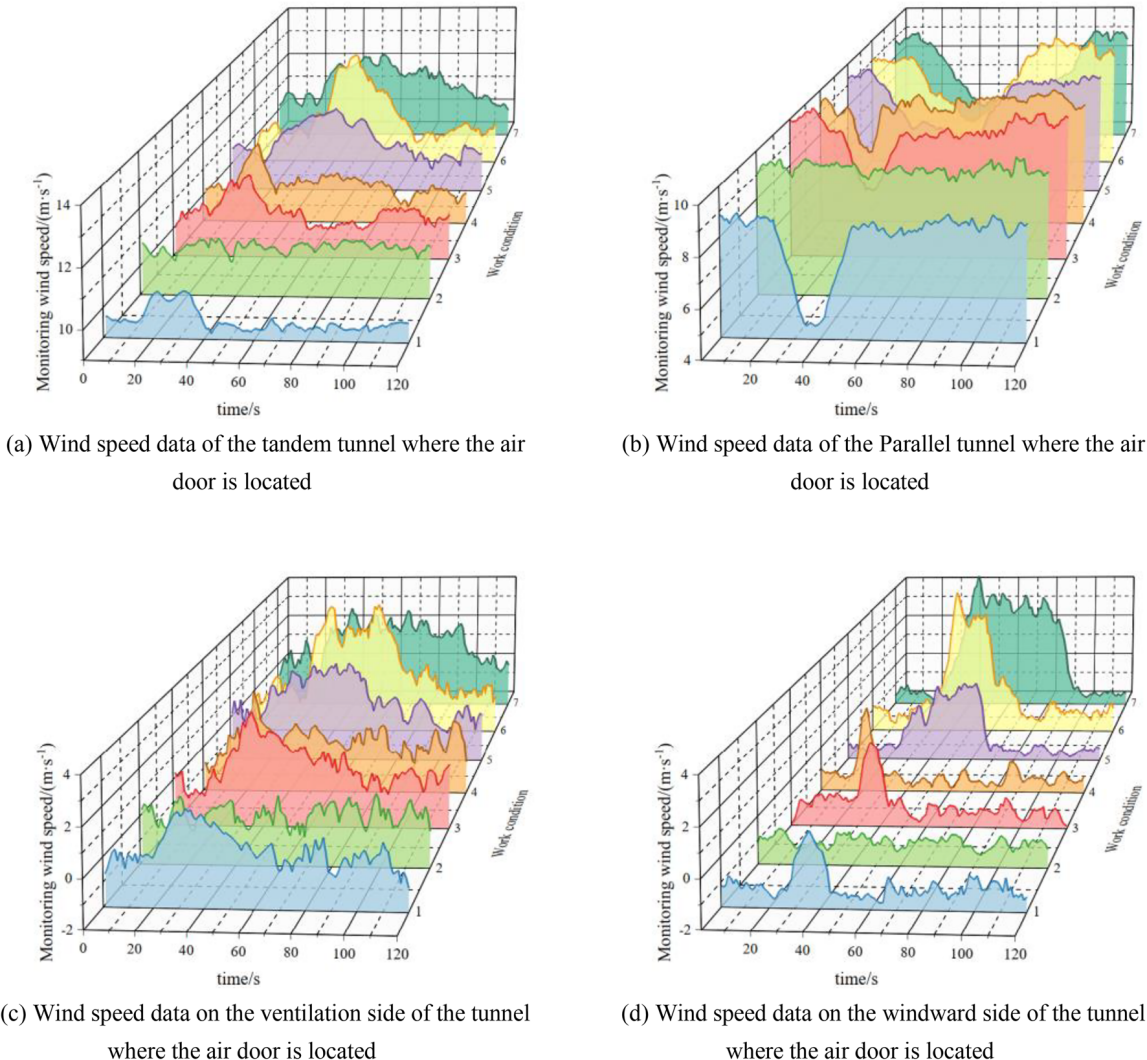
Variation in the wind speed data at each position with different parameters.

Some of the working conditions are shown in Table 4, and their data are displayed in Fig. 9.

**Table 4:** Setting of each parameter condition.

Work condition	Parameter 1	Parameter 2 (s)	Parameter 3	Parameter 4 (m/s)
1	3°/s	5	60°	9.5
2	0°/s	0	0°	10.5
3	3°/s	5	60°	10.5
4	10°/s	5	60°	10.5
5	3°/s	30	60°	10.5
6	3°/s	5	90°	10.5
7	10°/s	30	90°	10.5

According to Fig. 9, the abnormal fluctuation time and amplitude of the wind speed data at each measurement point under different working conditions are different, but the trend is consistent with that of the flow field in the numerical simulation.

### Evaluation indices of time identification and stage division

To verify the identification effect of the proposed method on the air door opening and closing time, three indicators, namely, the accuracy ($AC$), precision ($PR$), and recall ($RE$), were selected. The average value of these indicators in every air door opening and closing stage was taken to evaluate the effect of the opening and closing stage division. These were calculated using the numbers of true positives ($TP$), true negatives ($TN$), false negatives ($FN$), and false positives ($FP$). True positive (TP) refers to instances correctly identified as the event of interest. True negative (TN) refers to instances correctly identified as not being the event of interest. False negative (FN) refers to instances where the event of interest is not identified when it should have been. False positive (FP) refers to instances incorrectly identified as the event of interest when they are not. Equations (14)–(16) are the expressions used in these calculations.

14 $$ AC = \frac{TP + TN}{{TP + FP + TN + FN}} $$

15 $$ PR = \frac{TP}{{FP + TP}} $$

16 $$ SE = \frac{TP}{{TP + FN}} $$

### Experimental studies

In this section, 960 experimental data points representing 240 working conditions and 4 speed measurement points were used as the dataset. Fifty per cent of the dataset was used as the training set for the classification model, 30% was used as the training set for the regression model, and 20% was used as the test set for the overall method. To avoid any experimental bias, 10 cross-validations of the method effect were performed after each parameter change.

A comparison of the effects of time identification and stage classification of the ten model combinations was conducted to select the optimal combination of the classification and regression models. Among the ten model combinations, the classification models that were used were SVM^33^, random forest (RF)^34^, gradient boosting decision tree (GBDT)^35^, Bayesian network (BN)^36^, and backpropagation neural network (BPNN)^37^. The LASSO^38^ and elastic net regression (ENR)^39^ regression models were used.

**Figure 10:**
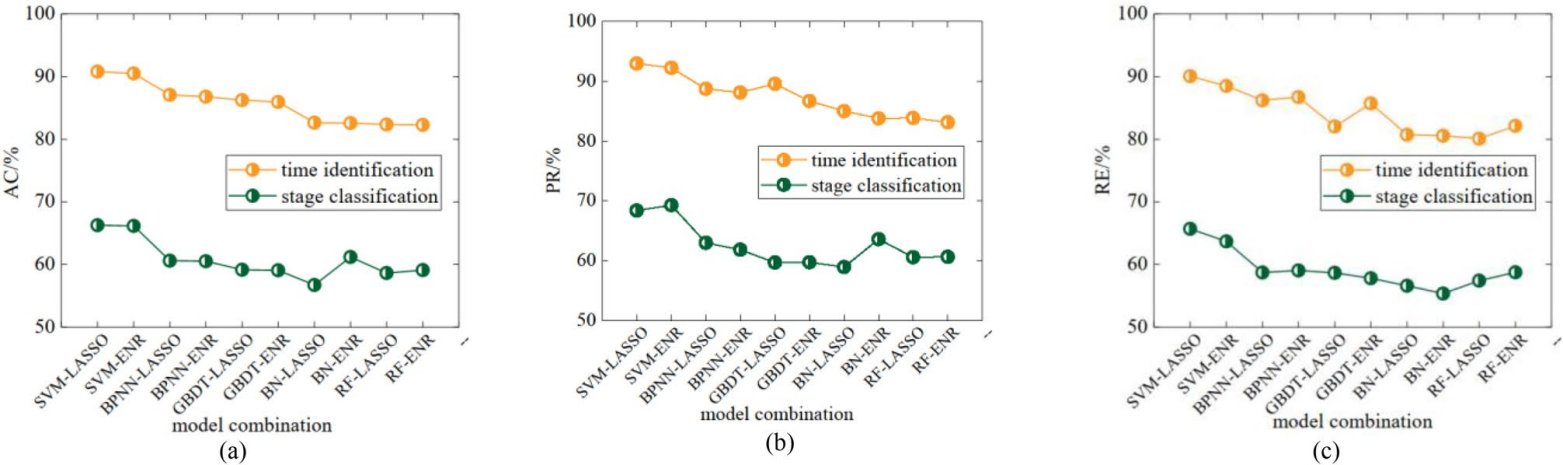
Individual indicator comparative analysis of different method combinations when the number of sliding window scales is 8.

A comparison of each indicator is shown in Fig. 10. The accuracy, precision, and recall of this method for air door opening and closing time identification and stage division are optimal when using the SVM model for classification and the LASSO model for regression.

**Figure 11:**
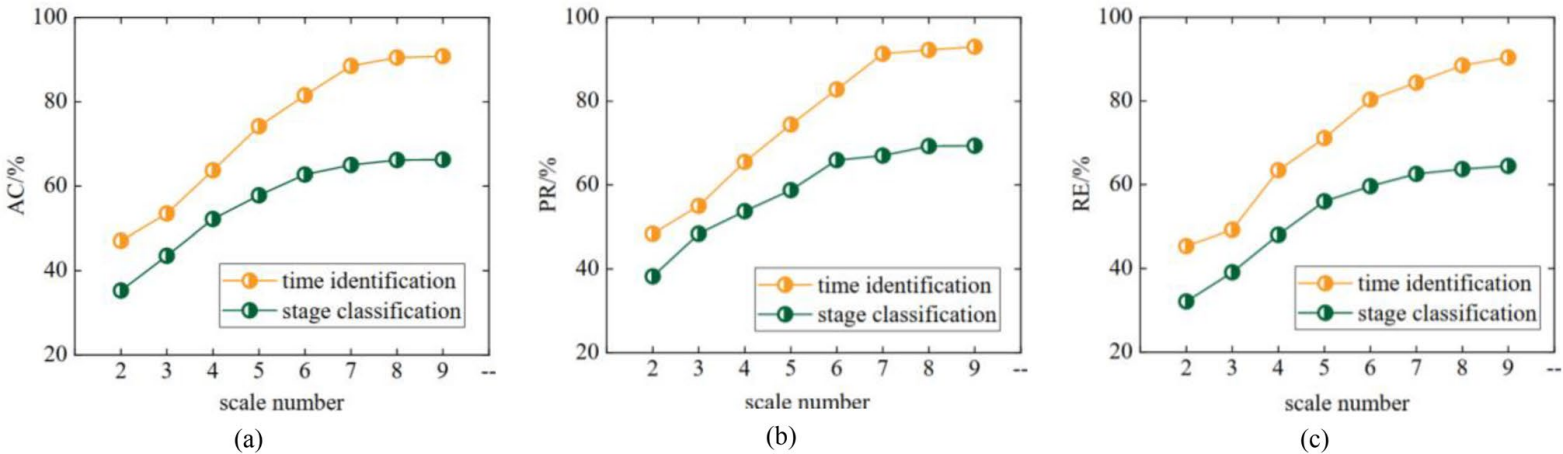
Individual indicator comparative analysis of different sliding window scale numbers when the method combination is SVM-LASSO.

To determine the optimal number of sliding windows, the effects of the method when the sliding window scale varied from 1 to 9 were compared. A comparison of all the indicators is shown in Fig. 11. The results show that when the number of sliding windows reaches 8, the accuracy, precision, and recall reach stability. Therefore, the optimal number of sliding windows is 8.

**Figure 12:**
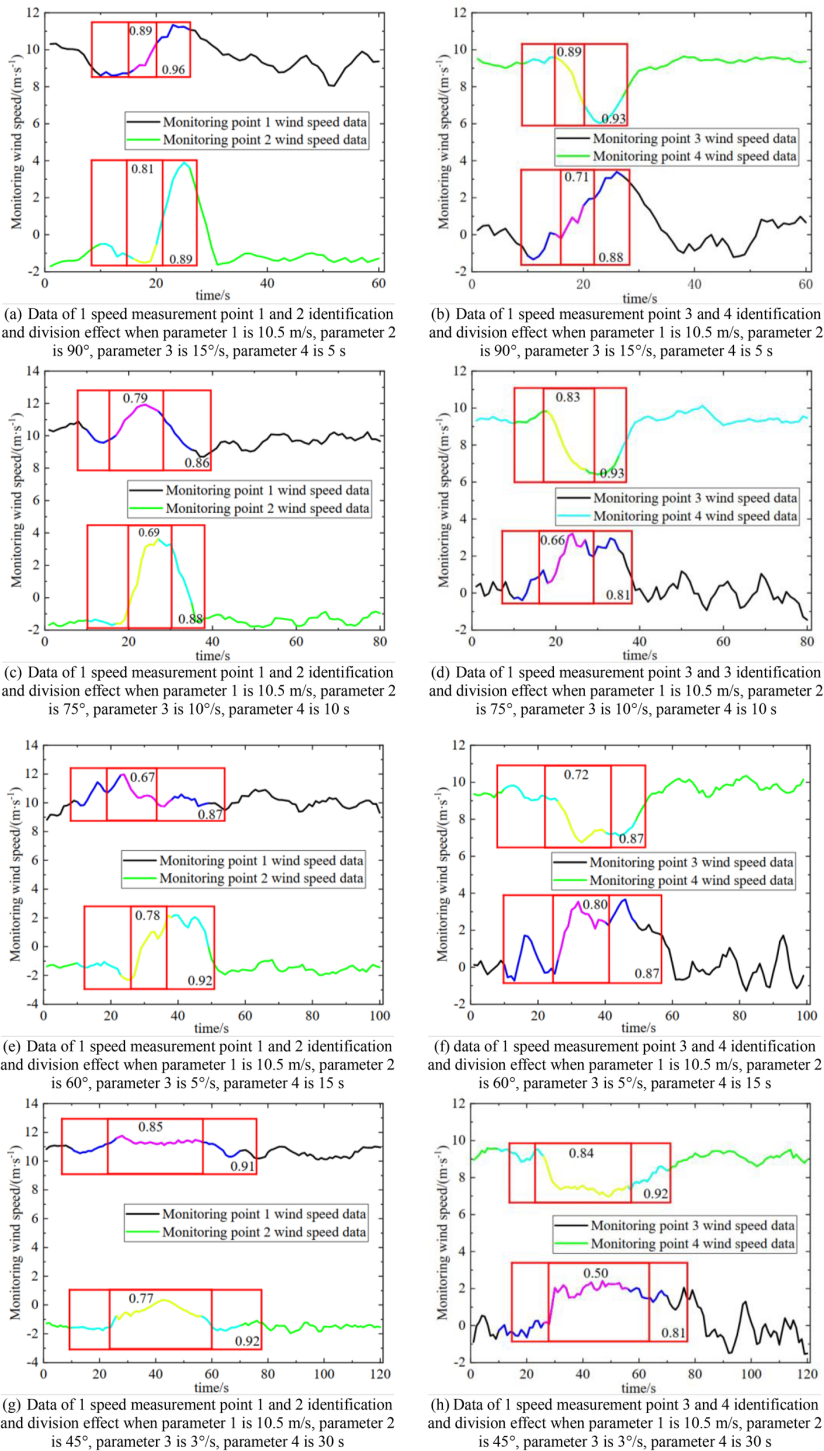
Part of the working conditions wind speed data time for air door opening and closing recognition and stage division effect diagram.

Through the above work, the optimal parameters of the method were obtained for air door opening and closing time identification and stage division. The accuracy, precision, and recall rate of the method using the optimal parameters for air door opening and closing time identification and stage division are above 90% and 62%, respectively. Figure 12 shows the effect of the method on the time identification and phase classification for some data.

## Conclusion

In this paper, a numerical simulation of the air door opening and closing process was conducted. The simulation results suggest the applicability of the proposed method in identifying the time of air door opening and closing, which can further be used for dividing the stages. Furthermore, this method was verified using an experimental approach. This can assist in realizing a basic theory for intelligent mine ventilation.

The proposed method in this study has four important implications. First, the method is based on single wind speed sensor data for air door opening and closing time identification and stage division, using less information to obtain more data while reducing the dependence on both the number and location of sensors. Second, the proposed feature extraction method, which is based on the DWT and statistical methods, can mine local fluctuation information and global information for subtime series data, and the extracted features are rich and interpretable. Third, the sequence of steps that need to be identified and then corrected can accurately solve the inconsistency between the air door opening and closing times and the flow field change time. Finally, with some additional improvements in the framework used in this method, it can be applied to the identification of other production activities that can cause abnormal fluctuations in wind speed monitoring data, such as mine car operation and cage hoisting.

The current method is suitable only for opening and closing air doors. Other production activities, time identification and stage division, and multiproduction activity classification are topics that should be considered in future investigations.

## Data Availability

A summary of the data used in this study is included in the paper, and a detailed data sample will be available upon request by contacting the corresponding author (201613602@sdtbu.edu.cn).
